# Prognostic Impact of EASIX According to the Timing of Assessment in Newly Diagnosed Multiple Myeloma Undergoing Autologous Stem Cell Transplantation

**DOI:** 10.3390/medsci14060659

**Published:** 2026-10-10

**Authors:** Diana Fortoeș, Ion Antohe, Vlad-Andrei Cianga, Cosmin-Daniel Minciună, Elena Nicorici, Elena Dolachi-Pelin, Mihaela Zlei, Mihaela Mentel, Loredana Dragoș, Iuliu Ivanov, Andrada Uhl, Ciprian Tomuleasa, Angela Dăscălescu

**Affiliations:** 1 Hematology Department, Regional Oncology Institute, 700483 Iasi, Romania; fortoes.diana.marina@email.umfiasi.ro (D.F.); vlad-andrei.cianga@email.umfiasi.ro (V.-A.C.); cosmin.minciuna@umfiasi.ro (C.-D.M.); elena.nicorici@iroiasi.ro (E.N.); elene.dolachi@iroiasi.ro (E.D.-P.); angela.dascalescu@umfiasi.ro (A.D.); 2 Grigore T. Popa University of Medicine and Pharmacy, 700115 Iasi, Romania; 3 Flow Cytometry Department, Regional Oncology Institute, 700483 Iasi, Romania; mihaela.zlei@iroiasi.ro (M.Z.); mihaela.mentel@iroiasi.ro (M.M.); 4 Molecular Diagnosis Department, Regional Institute of Oncology, 700483 Iasi, Romania; loredana.dragos@iroiasi.ro (L.D.); iuliuivanov@iroiasi.ro (I.I.); 5 Hematology Department, “Prof. Dr. Ion Chiricuta” Oncology Institute, 400015 Cluj-Napoca, Romania; anamaria.bancos@elearn.umfcluj.ro (A.U.); ciprian.tomuleasa@umfcluj.ro (C.T.); 6 Hematology Department, Iuliu Hațieganu University of Medicine and Pharmacy, 400347 Cluj-Napoca, Romania

**Keywords:** multiple myeloma, EASIX, endothelial dysfunction, autologous stem cell transplantation, overall survival, progression-free survival, prognostic biomarker, risk stratification

## Abstract

**Background/Objectives**: The Endothelial Activation and Stress Index (EASIX) is an easily accessible biomarker reflecting endothelial stress and has demonstrated prognostic value in several hematologic settings. However, its role in newly diagnosed multiple myeloma (NDMM), particularly according to the timing of assessment, remains insufficiently characterized. We evaluated the prognostic value of EASIX measured at diagnosis and immediately before autologous stem cell transplantation (autoSCT) for overall survival (OS) and progression-free survival (PFS). **Methods**: This retrospective single-center study included 99 transplant-eligible patients with NDMM who underwent autoSCT between 2017 and 2023. EASIX was calculated at diagnosis and pre-autoSCT and log_2_-transformed. Survival was assessed using Kaplan–Meier analysis and Cox proportional hazards regression. Optimal EASIX thresholds were explored using maximally selected log-rank statistics. Model discrimination was evaluated using Harrell’s concordance index and time-dependent area under the curve (AUC). **Results**: EASIX at diagnosis was available for 92 patients. Higher log_2_(EASIX) at diagnosis remained associated with inferior OS after adjustment for International Staging System (ISS) stage (HR 1.38, 95% CI 1.11–1.71; *p* = 0.0035). It also remained associated with shorter PFS after adjustment for ISS stage, age, and sex (HR 1.41, 95% CI 1.14–1.74; *p* = 0.001). Diagnosis EASIX demonstrated good discrimination for OS, with a C-index of 0.78 and sustained time-dependent AUC values. Conversely, pre-autoSCT EASIX was not significantly associated with OS or PFS when analyzed continuously, and cutoff-based analyses did not provide a consistent, robust prognostic signal. **Conclusions**: EASIX measured at diagnosis was associated with both OS and PFS in transplant-eligible NDMM, whereas continuous pre-autoSCT EASIX showed no consistent association with either outcome. A direct paired comparison demonstrated a significantly stronger prognostic association of diagnosis EASIX with PFS, while the corresponding between-timepoint difference for OS was not statistically significant. These findings support further evaluation of EASIX timing in larger independent cohorts.

## 1. Introduction

Multiple myeloma (MM) is a B-cell neoplasm characterized by the clonal expansion and proliferation of bone marrow (BM) plasma cells (PCs), that ultimately leads to end-organ damage [1]. The evolution to active MM from more indolent disease states, such as monoclonal gammopathy of undetermined significance (MGUS) and smoldering MM is driven by the progressive accumulation of genetic lesions in the PC genome. Longitudinal genomic studies have identified both relatively stable and evolution-driven patterns of progression [2,3]. Active, newly diagnosed MM (NDMM) displays marked genetic heterogeneity among distinct PC subclones, which is then dynamically shaped across the evolution of the disease by tumor genetic instability and selection pressure by sequential therapies and the immune system [4,5].

Tumor evolution is further accompanied by dynamic remodeling of the BM microenvironment. Reciprocal interactions among malignant plasma cells, stromal cells, immune cells, and endothelial cells promote tumor survival, immune evasion, and treatment resistance [6]. A characteristic feature of progression from precursor states to active disease is the development of an angiogenic BM phenotype. BM microvessel density increases across the MGUS-SMM-MM continuum and has been associated with disease activity and inferior survival in newly diagnosed disease [7,8,9]. Beyond angiogenesis, hypoxia, inflammatory cytokine signaling, oxidative stress, and cell-adhesion pathways contribute to endothelial activation and a prothrombotic microenvironment. In particular, increased circulating vascular cell adhesion molecule-1 (VCAM-1) has been associated with advanced disease and inferior overall survival (OS) in MM [10]. Endothelial dysfunction may therefore represent a clinically relevant component of MM biology, although its value as a prognostic marker and therapeutic target remains to be established.

The International Staging System (ISS), Revised ISS (R-ISS), and Second Revision of the ISS (R2-ISS) provide robust disease-specific risk stratification using tumor burden, biochemical variables, cytogenetic abnormalities, and lactate dehydrogenase (LDH) [11,12,13]. Nevertheless, these systems do not fully capture intratumoral heterogeneity or host-related vulnerability. Frailty assessment provides additional prognostic information in patients with newly diagnosed multiple myeloma and may help identify patients at increased risk of adverse clinical outcomes [14]. Accordingly, there is continued interest in simple biomarkers that integrate disease burden, organ dysfunction, and systemic biological stress beyond conventional staging systems.

The Endothelial Activation and Stress Index (EASIX), calculated as LDH (U/L) × creatinine (mg/dL)/platelet count (10^9^/L), was initially developed in recipients of allogeneic hematopoietic cell transplantation (allo-HCT) with acute graft-versus-host disease (GVHD) to predict OS and non-relapse mortality (NRM) [15]. Subsequent studies extended its evaluation to the preconditioning period and serial post-transplant timepoints, demonstrating associations with NRM, OS, fluid overload, acute GVHD, and sinusoidal obstruction syndrome [16,17,18,19,20,21]. Studies using direct endothelial biomarkers further support the concept that pre-existing endothelial vulnerability may influence susceptibility to subsequent transplant-related injury [22]. The EASIX score has also been evaluated outside allo-HCT, including lower-risk myelodysplastic syndromes, chimeric antigen receptor T-cell therapy, coronary artery disease, and COVID-19 [23,24,25,26,27]. These observations suggest that EASIX may capture clinically relevant systemic stress across several disease contexts; however, because its components also reflect cellular turnover, renal dysfunction, and thrombocytopenia, it should not be regarded as an endothelial-specific biomarker.

Evidence regarding the EASIX score in MM remains limited but is expanding. The EASIX measured at diagnosis has been associated with inferior OS in retrospective MM cohorts [28,29]. More recently, pre-transplant EASIX was associated with OS and progression-free survival (PFS) in an autologous stem cell transplantation (autoSCT) cohort, whereas another study examined its association with engraftment syndrome after autoSCT [30,31]. The prognostic implications of measuring EASIX at different phases of treatment within the same transplant-eligible cohort remain insufficiently characterized. We therefore aimed to evaluate the prognostic value of EASIX in patients with newly diagnosed MM (NDMM) undergoing autoSCT by assessing its association with OS and PFS when calculated at diagnosis and immediately before transplantation.

## 2. Materials and Methods

This observational, retrospective, single-center study included transplant-eligible patients with NDMM who underwent autoSCT at the Hematology Department of the Regional Oncology Institute in Iași between 2017 and 2023. Patients were diagnosed and received induction treatment either at our institution or at referring hematology centers. This study was approved by the institutional ethics committee “Comisia de etică a cercetării clinice a Institutului Regional de Oncologie din Iași”, approval code 1114/8 April 2019.

The International Staging System (ISS) was calculated at diagnosis. The EASIX score was calculated at diagnosis and pre-autoSCT (24 h before melphalan conditioning), as follows: LDH (U/L) × Creatinine (mg/dL)/platelet count (10^9^/L). Chromosomal abnormalities (CAs) were evaluated by conventional cytogenetic studies, multiplex ligation-dependent probe amplification (MLPA) or fluorescence in situ hybridization (FISH). In patients in whom FISH data was available, CA were classified, as per the revised ISS (R-ISS), as high- (del(17p), t(4;14), t(14;16), chromosome 1 anomalies), or standard-risk (all others). Patients in whom conventional cytogenetics and MLPA did not detect anomalies and with missing FISH data were classified as unknown cytogenetic risk.

Patients treated between 2017 and 2020 received VCD, while those treated from 2021 onward received the DVTD protocol following its reimbursement in Romania. The treatment regimens were administered following the standard dosage schedule approved by the European Medicines Agency and the Romanian Health Ministry National Protocol. Patients proceeded to autoSCT after achieving at least a partial response (PR). Patients who progressed while receiving first-line therapy underwent a second line of treatment and achieved at least a PR before proceeding to autoSCT.

### Statistical Analysis

Categorical and continuous variables were compared using the Pearson’s χ^2^ test and Fisher’s test when appropriate, and the Mann–Whitney U test, respectively. The primary endpoints of the study were overall survival (OS), defined as the interval from diagnosis to death from any cause or to the last follow-up date, and progression-free survival (PFS), defined as time from autoSCT to disease progression, death from any cause, or date of last follow-up, whichever occurred first. Response criteria were defined as per the International Myeloma Working Group recommendations. Patients alive without progression were censored at last follow-up. Overall survival probabilities were estimated using the Kaplan–Meier method, and survival distributions were compared using the log-rank test. EASIX values were log_2_ transformed, according to the literature data [15]. To determine the optimal EASIX cutoff in relation to survival outcomes, maximally selected log-rank statistics with exact Gauss *p*-value approximation were applied, consistent with previously published EASIX methodology [27] and implemented in the maxstat package, version 0.7.26 in R.

The relative risk of events for OS, along with the corresponding 95% confidence intervals (95% CI), was evaluated using Cox proportional hazards models in both univariate and multivariate analyses. The Cox proportional hazards model incorporated a log_2_-transformed EASIX index. To formally compare the prognostic effects of EASIX measured at diagnosis and pre-autoSCT, paired analyses were performed in patients with both measurements available. For PFS, both continuous log_2_(EASIX) measurements were simultaneously entered into a Cox proportional-hazards model. For OS, because pre-autoSCT EASIX was measured after diagnosis, the direct paired comparison was performed using autoSCT as the landmark and post-autoSCT survival as the time scale. The difference between the diagnosis and pre-autoSCT EASIX coefficients was assessed using a one-degree-of-freedom Wald contrast and confirmed by likelihood-ratio testing. ISS-adjusted models were performed as sensitivity analyses.

The discriminatory performance of the Cox model was assessed using the concordance index (C-index). To further evaluate the temporal stability of model performance, time-dependent discrimination analysis was performed using dynamic area under the curve (AUC) estimates. To account for potential optimism in model discrimination, internal validation was performed using 1000 bootstrap resamples. For each bootstrap sample, the Cox model was refitted and its discrimination was evaluated both in the bootstrap sample and in the original dataset. Optimism was calculated as the mean difference between bootstrap and test performance and subtracted from the apparent estimate. Harrell’s concordance index (C-index) and inverse-probability-of-censoring-weighted (IPCW) time-dependent AUCs at 18, 24, 36, 48, and 60 months were evaluated. Comparisons between the EASIX, ISS, and the combined EASIX + ISS model were performed on the same complete-case–cohort. A *p*-value < 0.05 was considered statistically significant for all analyses. All statistical analyses were performed using SPSS software (version 26) and R (version 4.5.2).

## 3. Results

**Figure 1:**
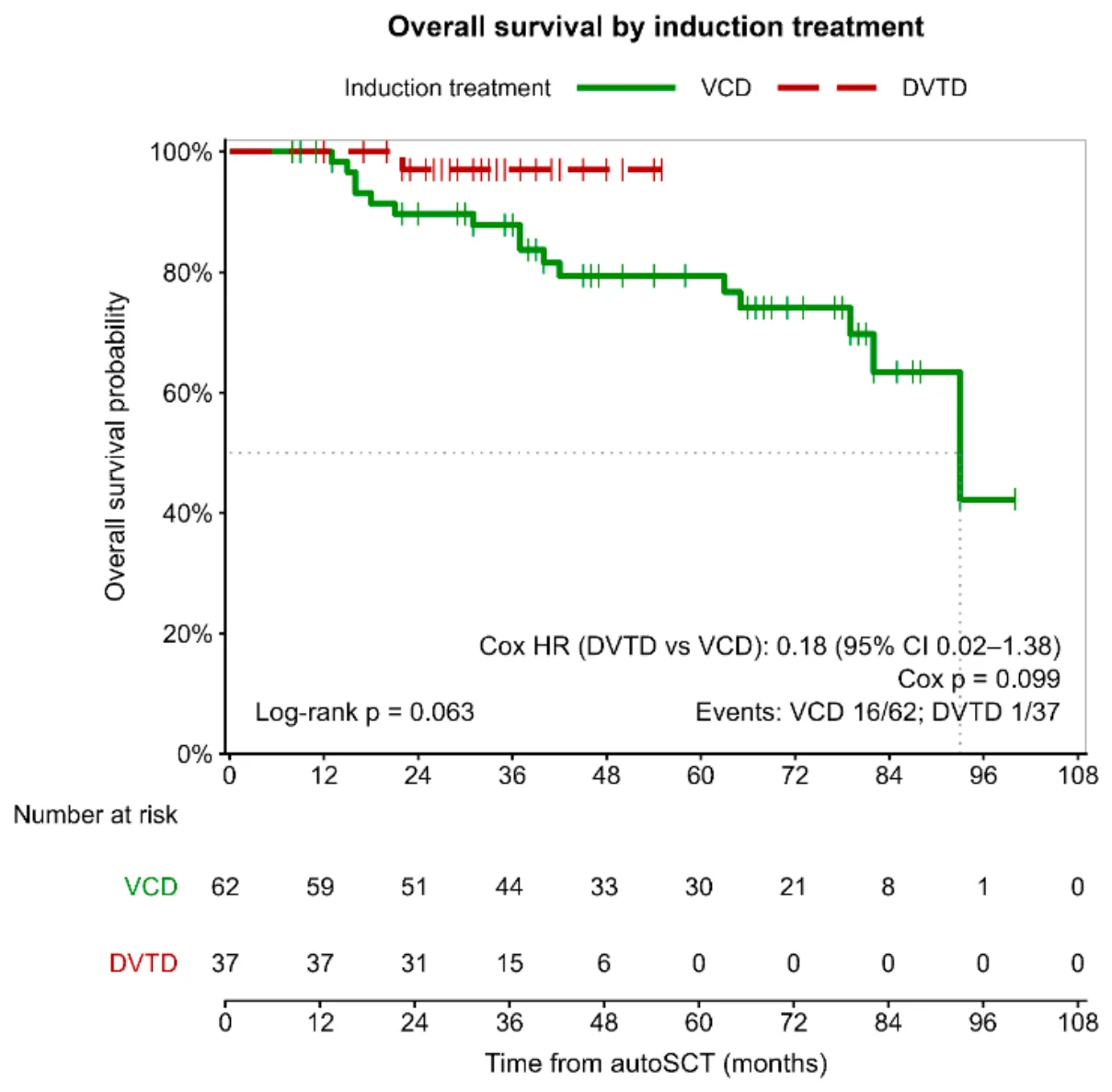
Overall survival after autoSCT according to induction regimen. Short vertical marks on the Kaplan–Meier curves indicate censored observations. Dotted horizontal and vertical lines indicate the 50% survival probability and the corresponding time point, respectively.

**Figure 2:**
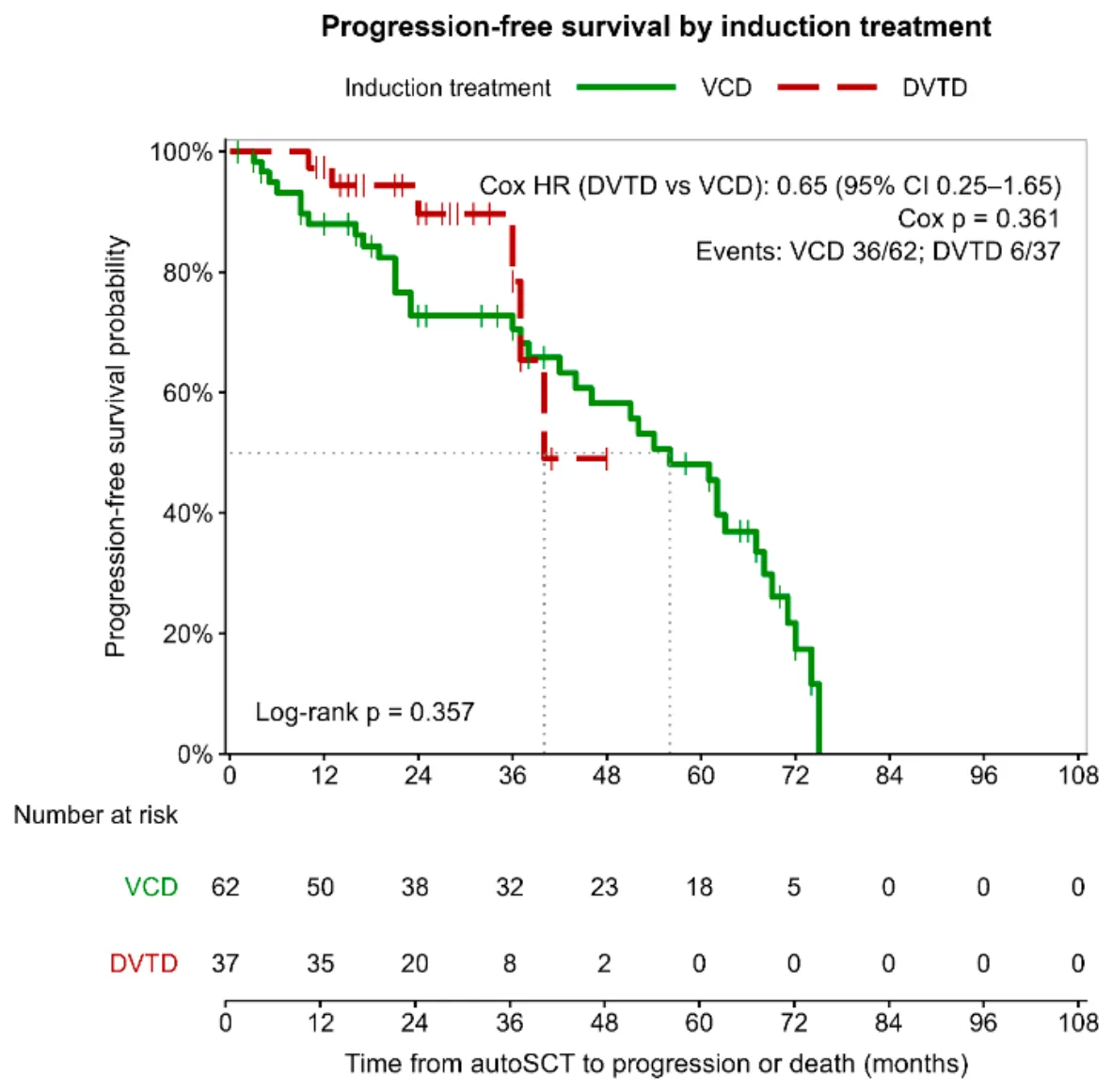
Progression-free survival after autoSCT according to induction regimen. Short vertical marks on the Kaplan–Meier curves indicate censored observations. Dotted horizontal and vertical lines indicate the 50% survival probability and the corresponding time point, respectively.

Our study included 99 patients with NDMM who received autoSCT at our center between 2017 and 2023. Patients were treated with either VCD (*n* = 62) or DVTD (*n* = 37) induction regimens. Demographic and clinical characteristics were comparable between the two induction regimens (Table 1). The median follow-up duration was 45 months for the entire cohort (95% CI: 38.2–51.7), 71 months (95% CI: 64.6–77.3) and 33 months (95% CI: 28.6–37.3) for the VCD and DVTD group, respectively. The median time from diagnosis to autoSCT was 8 months (IQR, 6–10; range, 4–29). In the overall cohort, 17 deaths and 42 progression events were recorded. The Kaplan–Meier estimated median OS was 93 months, while median PFS was 56 months. In the OS analysis stratified by induction regimen, the median OS was 93 months in the VCD group and was not reached in the DVTD group. However, only one death occurred among the 37 patients treated with DVTD, compared with 16 deaths among the 62 patients treated with VCD (Figure 1). The resulting hazard-ratio estimate was imprecise (HR 0.18, 95% CI 0.02–1.38) and did not demonstrate a statistically significant difference in OS between induction regimens by either Cox regression or the log-rank test (Table 2). For PFS, the median values were 56 months for VCD and 40 months for DVTD, with no statistically significant difference between induction regimens (Figure 2).

Treatment responses following induction are summarized in Table 3. Overall, rates of ≥VGPR were significantly higher in the DVTD group. Response distribution before and after autoSCT according to induction regimen is summarized in Table 4. Before autoSCT, CR was achieved in 15/62 patients (24.2%) treated with VCD and in 21/37 patients (56.8%) treated with DVTD. After autoSCT, CR was documented in 28/61 patients (45.9%) in the VCD group and in 31/37 patients (83.8%) in the DVTD group. Among patients without CR before autoSCT, conversion to CR after transplantation occurred in 18/47 patients (38.3%) receiving VCD and in 10/16 patients (62.5%) receiving DVTD. In univariate Cox analysis, response parameters, including complete response and ≥VGPR prior to transplantation, were not significantly associated with overall survival (HR 0.76, 95% CI 0.26–2.26; *p* = 0.620 and HR 1.28, 95% CI 0.40–4.09; *p* = 0.683, respectively).

### 3.1. EASIX and Survival Outcomes

In the following section, we evaluate the prognostic significance of the EASIX score calculated at diagnosis and immediately before conditioning, on overall survival and progression-free survival.

#### 3.1.1. Prognostic Impact of EASIX on Overall Survival

##### Prognostic Impact of EASIX Calculated at Diagnosis on OS

A total of 92 patients with NDMM had available EASIX values at diagnosis and were included in the survival analysis. During the follow-up period, 15 deaths were recorded. median OS in the diagnosis-EASIX cohort was 93 months (95% CI, not estimable); however, this estimate occurred in the sparsely populated tail of the Kaplan–Meier curve and should therefore be interpreted cautiously.

**Figure 3:**
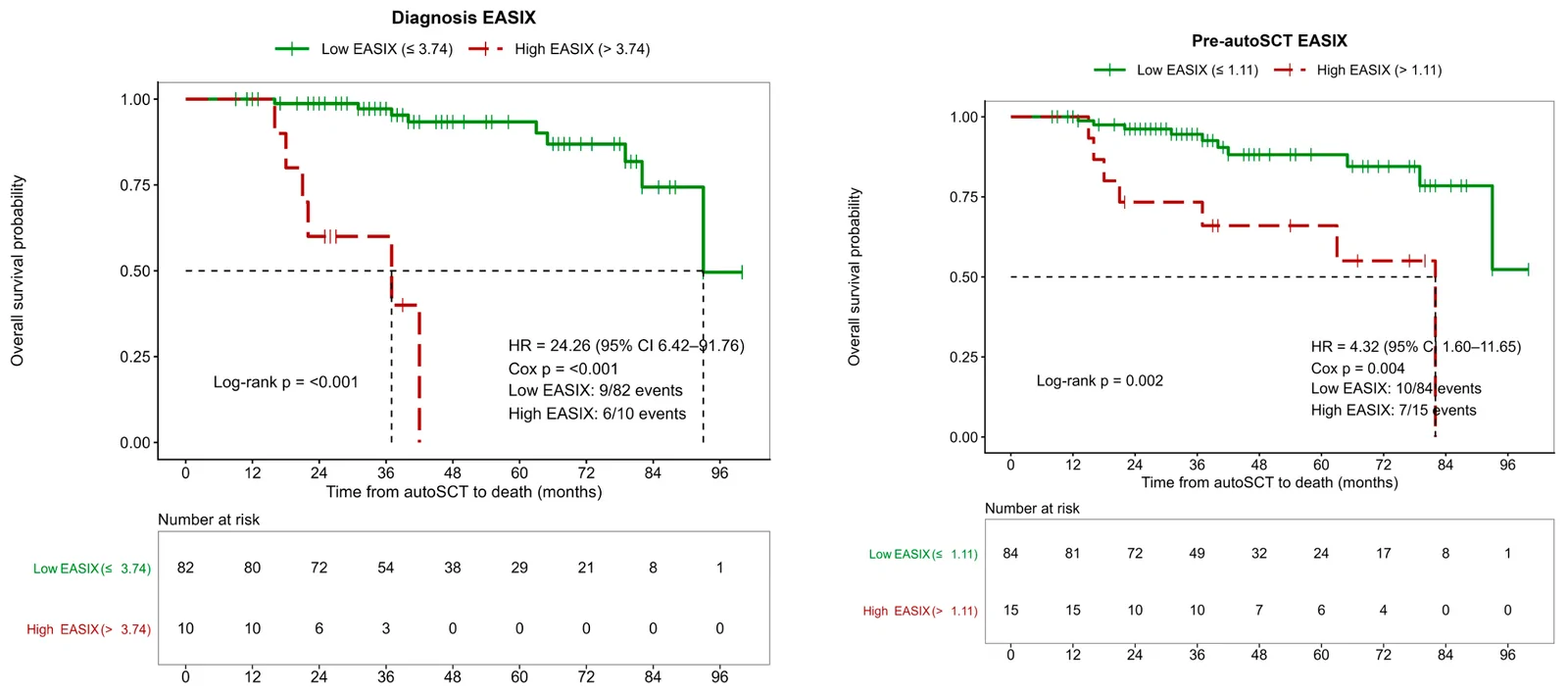
Kaplan–Meier curves for overall survival according to the EASIX at diagnosis and pre-autoSCT, using data-driven cutoffs. High-EASIX diagnosis was associated with inferior survival, whereas pre-autoSCT EASIX showed a weaker association. Short vertical marks on the Kaplan–Meier curves indicate censored observations. Dashed horizontal and vertical lines indicate the 50% survival probability and the corresponding estimated median progression-free survival time, respectively.

The analysis identified an optimal log_2_(EASIX) cutoff of 1.9, corresponding to an EASIX value of 3.74, which best discriminated patients according to overall survival (M = 4.46, *p* = 0.00020). Using this threshold, patients were stratified into low-EASIX (≤3.74) and high-EASIX (>3.74) groups. The main clinical characteristics of the two groups are presented in Table 5. Diagnosis EASIX was unavailable for 7 patients. Compared with the 92 patients included in the diagnosis-EASIX analysis, these patients showed no material differences in baseline age, sex, ISS stage, renal function, induction regimen, or observed survival outcomes, although comparisons were limited by the small number of excluded patients. Among the 92 patients included in the analysis, 82 patients (89.1%) were classified as low EASIX and 10 patients (10.9%) as high EASIX. The distribution of deaths across groups showed that 9 deaths (11%) occurred in the low-EASIX group and 6 deaths (60%) in the high-EASIX group. This difference translated into a clear separation of survival curves. Patients with high EASIX (>3.74) experienced inferior survival, with a median OS of 37 months (95% CI, 21 months–not estimable), whereas median OS was not reached in the low-EASIX group (95% CI, 93 months–not estimable; log-rank *p* < 0.001) (Figure 3, Table 6).

In univariable Cox regression using the categorical variable, high EASIX was associated with a markedly increased hazard of death (HR 24.26, 95% CI 6.42–91.76; *p* < 0.001). the proportional hazards assumption was not violated (Schoenfeld residual test, *p* = 0.537). However, this estimate should be interpreted cautiously because the high-EASIX subgroup included only 10 patients, with 6 deaths, resulting in a wide confidence interval. When analyzed as a continuous variable in univariable Cox regression, higher log_2_(EASIX) at diagnosis was significantly associated with inferior OS (HR 1.41, 95% CI 1.15–1.72; *p* = 0.0007).

In multivariable Cox regression analysis adjusted for ISS stage (*n* = 79, 15 events), log_2_(EASIX) remained associated with OS after adjustment for ISS stage (HR 1.38, 95% CI 1.11–1.71; *p* = 0.0035). ISS stage was not significantly associated with OS in this cohort. In a sensitivity analysis additionally adjusted for age, the prognostic effect of EASIX remained stable (HR 1.36, 95% CI 1.10–1.69; *p* = 0.0042) (Table 7). The model demonstrated good discriminatory ability, with a Harrell’s concordance index (C-index) of 0.784 (SE = 0.054), although should be interpreted cautiously because of the limited number of deaths relative to the number of model parameters.

**Figure 4:**
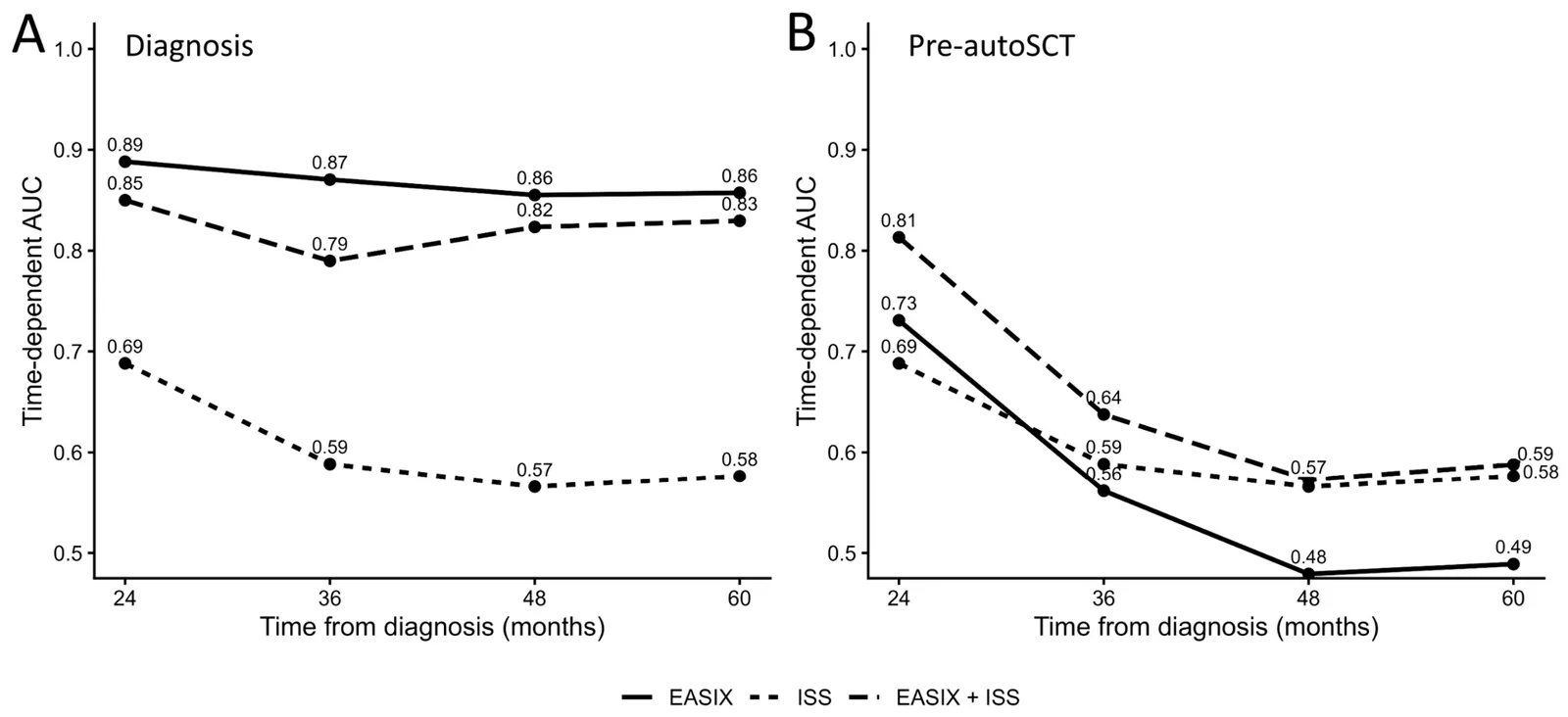
Time-dependent area under the curve (AUC) for overall survival according to EASIX measured at diagnosis (**A**) and pre-autoSCT (**B**). Models based on the EASIX, ISS, and the combined EASIX + ISS model are shown.

In terms of predictive performance, the EASIX-based model showed good and sustained discrimination over time. The apparent Harrell C-index was 0.78, and internal validation with 1000 bootstrap resamples demonstrated minimal optimism, with an optimism-corrected C-index of 0.78. Optimism-corrected time-dependent AUC values remained consistently high across the evaluated time points, ranging from 0.83 to 0.89 between 18 and 60 months (Figure 4A). In the common complete-case–cohort (*n* = 79), the optimism-corrected C-indices were 0.80 for EASIX alone, 0.58 for ISS alone, and 0.75 for the combined EASIX + ISS model. These findings indicate that adding ISS did not provide additional discriminatory benefit beyond the EASIX score alone in this cohort.

##### Prognostic Impact of EASIX Calculated Pre-AutoSCT on OS

Pre-autoSCT EASIX was available for all 99 patients, among whom 17 deaths were recorded. The optimal cutoff for log_2_pre-autoSCT EASIX was identified at 0.15 (corresponding to untransformed EASIX of approximately 1.11) using maximally selected log-rank statistics (M = 3.11, *p* = 0.024). Using this threshold, 84 patients (84.8%) were classified as low EASIX and 15 patients (15.2%) as high EASIX. Deaths occurred in 10 of 84 patients (11.9%) in the low-EASIX group and 7 of 15 patients (46.7%) in the high-EASIX group. Kaplan–Meier analysis demonstrated inferior survival in the high-EASIX subgroup (log-rank *p* = 0.0024) (Figure 3, Table 6). This was confirmed in univariable Cox regression, where high EASIX was associated with worse overall survival (HR 4.13; 95% CI 1.53–11.14; *p* = 0.005).

However, when analyzed as a continuous variable, pre-transplant log_2_(EASIX) was not associated with overall survival (HR 1.26; 95% CI 0.80–1.99; *p* = 0.325). This remained non-significant after adjustment for ISS (HR 1.18; *p* = 0.501) and in a sensitivity model including age (HR 1.20; 95% CI 0.74–1.93; *p* = 0.459). In the fully adjusted grouped model including age and ISS, high pre-transplant EASIX remained independently associated with inferior overall survival (HR 4.40; 95% CI 1.50–12.92; *p* = 0.007). The proportional hazards assumption was satisfied in all models. Model discrimination was modest, with a C-index of 0.61 for the continuous EASIX and 0.69 for the combined EASIX + ISS model.

Time-dependent AUC analysis (Figure 4) showed moderate early discrimination (AUC 0.80 at 18 months, 0.73 at 24 months), followed by a marked decline to approximately 0.48–0.56 beyond 36 months. The ISS model showed more stable performance over time, and the combined model did not provide sustained improvement. Overall, pre-autoSCT EASIX appears to have limited and non-durable prognostic value for OS, and the observed cutoff-based association may reflect a data-driven threshold effect in a small subgroup.

#### 3.1.2. Prognostic Impact of the EASIX on Progression-Free Survival

##### Prognostic Impact of EASIX Calculated at Diagnosis on PFS

**Figure 5:**
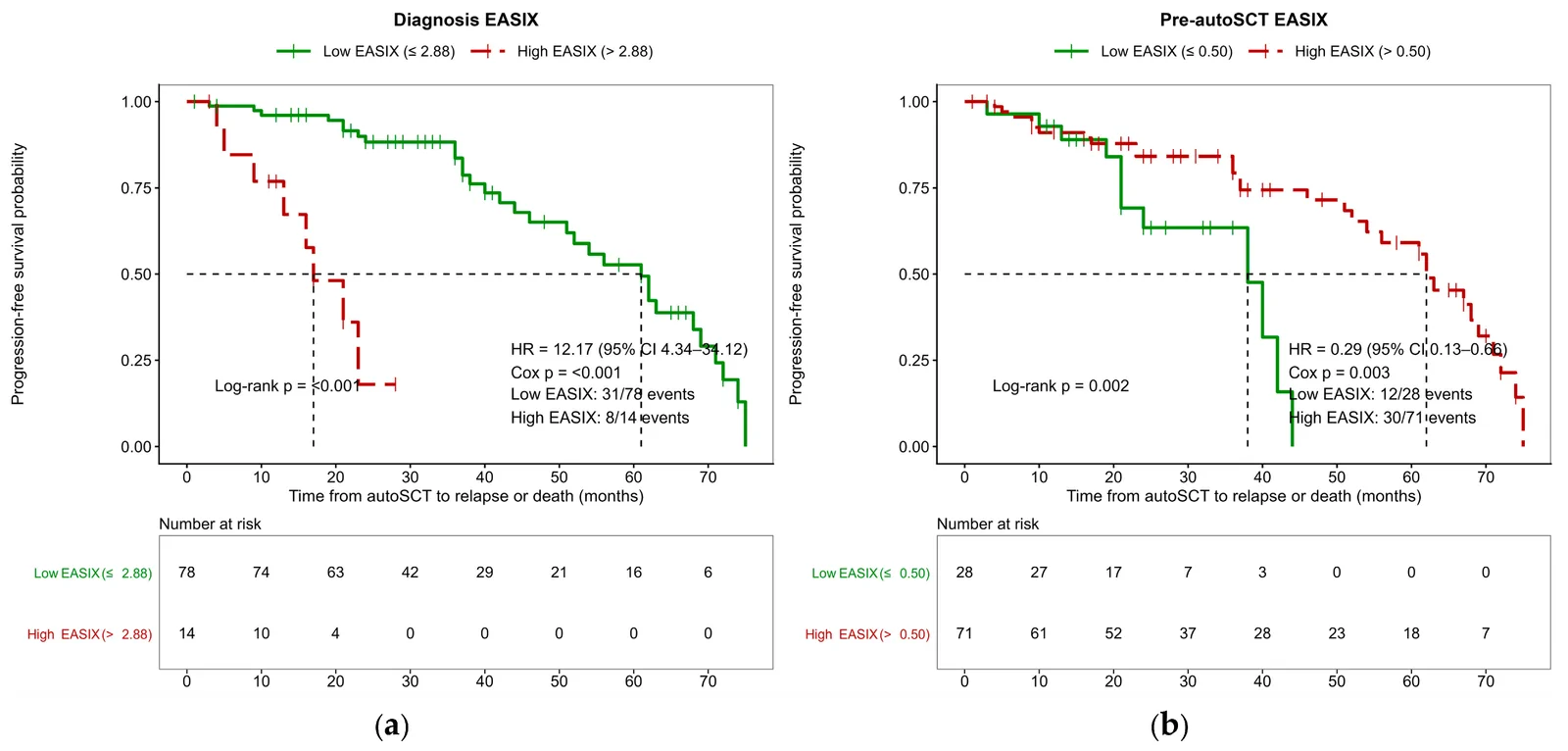
Prognostic impact of EASIX at diagnosis and pre-transplant on progression-free survival. Kaplan–Meier curves evaluating the association between EASIX and progression-free survival (PFS) in patients with multiple myeloma undergoing autologous stem cell transplantation (autoSCT). (**a**) Kaplan–Meier estimates of PFS according to EASIX at diagnosis, dichotomized using the optimal cut-off derived from maximally selected rank statistics. (**b**) Kaplan–Meier estimates of PFS according to pre-transplant EASIX using the corresponding cut-off. Short vertical marks on the Kaplan–Meier curves indicate censored observations. Dashed horizontal and vertical lines indicate the 50% survival probability and the corresponding estimated median progression-free survival time, respectively.

EASIX at diagnosis was available for 92 patients, among whom 39 progression-free survival (PFS) events were recorded. Maximally selected log-rank analysis identified a log_2_(EASIX) cutoff of 1.53, corresponding to an original EASIX value of 2.88. Using this threshold, 78 patients were classified in the low-EASIX group and 14 in the high-EASIX group. The selected cutoff remained statistically significant after correction for data-driven cutoff selection, with an exact-Gauss-adjusted *p*-value of 0.035 and a conditional Monte Carlo-adjusted *p*-value of 0.032. Median PFS was 61 months in the low-EASIX group and 17 months in the high-EASIX group, with a conventional log-rank *p*-value of <0.001 (Figure 5a) (Table 8).

In the univariable cutoff-based Cox model, a high EASIX at diagnosis was associated with a markedly increased hazard of progression or death compared with a low EASIX (HR, 12.17; 95% CI, 4.34–34.12; *p* < 0.001). When EASIX was analyzed continuously on the log_2_ scale, each doubling of EASIX at diagnosis was associated with a 41% increase in the hazard of progression or death (HR, 1.41; 95% CI, 1.16–1.71; *p* < 0.001). This association remained significant after adjustment for ISS stage, age, and sex, both in the cutoff-based model (adjusted HR for high versus low EASIX, 11.62; 95% CI, 3.42–39.45; *p* < 0.001) and in the continuous model (adjusted HR per EASIX doubling, 1.41; 95% CI, 1.14–1.74; *p* = 0.001) (Table 9).

Overall, EASIX at diagnosis showed a consistent and independent association with inferior PFS, both as a cutoff-defined variable and as a continuous log_2_-transformed marker.

##### Prognostic Impact of EASIX Calculated Pre-AutoSCT on PFS

Pre-autoSCT EASIX was available for all 99 patients, among whom 42 PFS events occurred. Maximally selected log-rank analysis identified a log_2_(EASIX) cutoff of −1.00, corresponding to an original EASIX value of 0.50. This threshold classified 28 patients in the low-EASIX group and 71 in the high-EASIX group. Unexpectedly, the high-EASIX group showed longer Median PFS than the low-EASIX group (62 vs. 38 months; conventional log-rank *p* = 0.002). (Figure 5b). However, this cutoff did not remain statistically significant after adjustment for data-driven cutoff selection, with an exact-Gauss-adjusted *p*-value of 0.188 and a conditional Monte Carlo-adjusted *p*-value of 0.180 (Table 8).

In the cutoff-based Cox model, high pre-autoSCT EASIX was associated with a lower hazard of progression or death compared with low pre-autoSCT EASIX in both univariable analysis (HR, 0.29; 95% CI, 0.13–0.66; *p* = 0.003) and after adjustment for ISS stage, age, and sex (adjusted HR, 0.21; 95% CI, 0.09–0.51; *p* < 0.001). However, this association was not supported when pre-autoSCT EASIX was analyzed as a continuous variable. In the continuous Cox model, pre-autoSCT EASIX was not significantly associated with PFS in either univariable analysis (HR per doubling, 0.84; 95% CI, 0.56–1.24; *p* = 0.372) or adjusted analysis (adjusted HR per doubling, 0.74; 95% CI, 0.47–1.18; *p* = 0.204) (Table 9). The paradoxical direction of the cutoff-based estimate was therefore considered a data-driven artifact and was not interpreted as evidence of a protective effect of higher pre-autoSCT EASIX.

##### Direct Comparison of the EASIX Measurement Timepoints

To formally assess whether the prognostic association of the EASIX score differed according to the timepoint of measurement, paired joint Cox models including both diagnosis and pre-autoSCT log_2_(EASIX) were fitted. For PFS (*n* = 92; 39 events), the prognostic effect of EASIX at diagnosis was significantly stronger than that of pre-autoSCT EASIX (ratio of HRs 2.03, 95% CI 1.21–3.41; Wald *p* = 0.008; likelihood-ratio *p* = 0.005). This difference remained significant after adjustment for ISS (*n* = 79; 36 events; ratio of HRs 2.00, 95% CI 1.17–3.43; Wald *p* = 0.011; likelihood-ratio *p* = 0.006). In contrast, in the autoSCT-landmark analysis of OS (*n* = 92; 15 deaths), the difference between the two EASIX coefficients was not statistically significant (ratio of HRs 1.34, 95% CI 0.72–2.48; Wald *p* = 0.359; likelihood-ratio *p* = 0.332), with similar results after adjustment for ISS.

## 4. Discussion

Despite substantial therapeutic progress, outcomes among patients with NDMM remain heterogeneous. The incorporation of anti-CD38 monoclonal antibodies into proteasome inhibitor- and immunomodulatory drug-based induction has improved response depth and PFS in transplant-eligible patients [32,33]. Nevertheless, access to quadruplet induction and subsequent cellular or bispecific antibody therapies remains uneven, with documented geographic and racial disparities in access to clinical trials [34]. In this setting, inexpensive and widely available prognostic biomarkers may help identify patients with adverse outcomes who require closer surveillance and prospective evaluation in risk-adapted strategies. However, a prognostic association alone does not establish that a biomarker predicts benefit from treatment intensification, which requires demonstration of a biomarker-by-treatment interaction [35].

In this retrospective cohort of patients with NDMM undergoing autoSCT, the EASIX score calculated at diagnosis was independently associated with both PFS and OS, whereas EASIX measured immediately before autoSCT was not prognostic. These findings indicate that the information captured by EASIX is time-dependent and may vary according to disease phase, treatment exposure, and patient selection.

At diagnosis, EASIX showed sustained apparent discrimination for OS, with time-dependent area under the curve values of 0.85–0.89 between 24 and 60 months. In this cohort, these estimates were numerically higher than those obtained with ISS alone. Adding ISS to the EASIX score did not materially increase apparent discrimination, suggesting overlapping between the prognostic information conveyed by the two measures. Although the initially observed discrimination raised concern regarding possible overoptimism given the limited number of OS events, bootstrap internal validation showed only minimal optimism for the continuous EASIX model. Moreover, in the common complete-case–cohort, incorporation of ISS did not improve optimism-corrected discrimination compared with the EASIX score alone, suggesting limited incremental discriminatory information from ISS in this dataset. Given the small number of deaths, however, this finding should be considered exploratory. External validation is required before concluding that EASIX performs better than established staging systems.

The association between the EASIX score at diagnosis and outcome is biologically plausible, but its mechanism is likely multifactorial. The angiogenic remodeling of the BM, endothelial activation, and increased expression of adhesion molecules are recognized features of active MM [7,8,9,10]. At the same time, each component of the EASIX score has potential non-endothelial determinants: LDH may reflect cellular turnover and aggressive disease biology, creatinine captures renal dysfunction and myeloma-related organ damage, and thrombocytopenia may reflect BM infiltration, treatment-independent host factors, or advanced disease. EASIX may therefore integrate tumor burden, organ dysfunction, marrow reserve, and systemic endothelial stress rather than provide a specific measurement of endothelial injury.

In contrast, pre-autoSCT EASIX was not associated with survival in either continuous or multivariable analyses, and maximally selected rank statistics did not identify a stable prognostic cutoff. Several factors may explain this discrepancy between timepoints. Patients who reach autoSCT represent a selected population with treatment-responsive or clinically stable disease, and induction therapy may attenuate the biological heterogeneity present at diagnosis. In addition, contemporary autoSCT for MM is associated with low early treatment-related mortality, while long-term outcomes are predominantly determined by relapse and disease progression rather than by severe transplant-related endothelial complications [36,37]. Consequently, an index partly influenced by systemic and endothelial stress may have limited discrimination when measured immediately before a generally well-tolerated autologous procedure.

This finding also highlights the biological differences between autologous and allogeneic transplantation. In allo-HCT, endothelial injury is implicated in several major causes of early morbidity and mortality, including acute GVHD, sinusoidal obstruction syndrome, fluid overload, and other endothelial complications. In that setting, the EASIX score has shown reproducible associations with NRM and survival when measured before conditioning and dynamically after transplantation [15,16,17,18,19,20,21]. The lower burden of immune-mediated and endothelial complications after autoSCT may reduce the contribution of the pathways captured by EASIX to subsequent survival.

Our findings extend the limited literature on the EASIX score in MM. Song et al. and Gursoy et al. reported inferior OS among patients with higher EASIX values measured at or near diagnosis [28,29], consistent with the association observed in our cohort. Importantly, our study also identified an association with PFS and evaluated the EASIX score at two clinically relevant timepoints within the same population. However, our negative pre-autoSCT result contrasts with the recently published study by Gullu Koca et al., in which higher log-transformed pre-transplant EASIX was independently associated with inferior OS among 274 autoSCT recipients; shorter PFS was also observed using an exploratory cutoff [30]. Differences in cohort size, number of events, timing of laboratory assessment, disease status at transplantation, induction and maintenance exposure, and multivariable model specification may account for the discordance. The study by Tolosa-Ridao et al., which associated post-apheresis EASIX with engraftment syndrome, further suggests that the clinical meaning of EASIX may depend on the exact sampling time and endpoint evaluated [31].

The cutoff analyses should also be interpreted as exploratory. In particular, the paradoxical cutoff-based association between higher pre-autoSCT EASIX and longer PFS likely reflects the instability of outcome-derived dichotomization in a modest-sized cohort rather than a biologically meaningful protective effect. Although maximally selected rank statistics account for the repeated search across candidate thresholds [38], outcome-derived cutpoints may still yield unstable effect estimates and do not establish a universally applicable clinical threshold. Importantly, the inverse association observed for pre-autoSCT EASIX was not supported by the continuous analyses and the selected cutoff did not remain significant after adjustment for data-driven cutoff selection. Accordingly, we prioritized continuous log_2_(EASIX) models for inference rather than dichotomization. Similarly, any cutoff derived at diagnosis requires validation in an independent cohort before clinical use.

From a clinical perspective, the EASIX score has several practical advantages: it is inexpensive, reproducible, and based on routinely available laboratory measurements. Its association with PFS and OS at diagnosis suggests that it could complement established disease-specific risk assessment, particularly when complete cytogenetic testing is unavailable. Nevertheless, the present data demonstrate prognostic rather than predictive value. Whether the EASIX score can identify patients who derive greater benefit from intensified induction, consolidation, maintenance, or closer supportive monitoring should be evaluated prospectively in studies designed to assess treatment-effect modification.

Several limitations should be acknowledged. First, the retrospective design introduces potential selection bias because only patients who retained eligibility for autoSCT were included; patients with early progression, severe toxicity, or declining performance status before transplantation were excluded. Second, the modest sample size and limited number of OS events may have reduced the stability of multivariable estimates and increased the risk of optimism in discrimination measures and cutoff selection. Third, incomplete cytogenetic data precluded consistent application of contemporary staging systems such as R-ISS and R2-ISS, preventing direct assessment of the incremental value of EASIX beyond comprehensive cytogenetic risk. Fourth, heterogeneity in follow-up, induction and maintenance exposure, and post-relapse therapy may have influenced survival outcomes. Fifth, direct markers of endothelial injury were unavailable, precluding mechanistic validation of the EASIX score as a surrogate of endothelial dysfunction. Finally, the absence of an external validation cohort limits the generalizability of the observed associations and any outcome-derived cutoff.

## 5. Conclusions

In this retrospective cohort of patients with multiple myeloma undergoing autologous stem cell transplantation, higher EASIX values at diagnosis were independently associated with shorter progression-free and overall survival. By contrast, the EASIX score measured immediately before autoSCT showed no consistent association with either outcome in continuous or dichotomized analyses.

These findings suggest that the prognostic information captured by EASIX may be timepoint-dependent. The EASIX score measured at diagnosis was associated with both OS and PFS, whereas continuous pre-autoSCT EASIX did not show a consistent prognostic association. Importantly, direct paired comparison demonstrated a significantly stronger association of diagnosis EASIX with PFS, while the corresponding between-timepoint difference for OS was not statistically significant. At diagnosis, the EASIX score may integrate overlapping dimensions of disease burden, cellular turnover, renal dysfunction, marrow reserve, and systemic or endothelial stress. Its prognostic signal may subsequently be attenuated by induction therapy and by the clinical selection of treatment-responsive patients who remain eligible for transplantation. Nevertheless, these mechanisms were not evaluated directly and should be considered hypothesis-generating.

Our results support further evaluation of the EASIX score at diagnosis as a readily available candidate prognostic biomarker in transplant-eligible newly diagnosed multiple myeloma. External validation using standardized measurement windows, continuous modeling, and adjustment for contemporary disease-risk systems is required before its incorporation into routine risk stratification. Future studies should also determine whether longitudinal changes in the EASIX score between diagnosis and transplantation provide incremental prognostic information beyond the baseline EASIX and established risk factors.

## Figures and Tables

**Table 1:** Demographic and clinical characteristics according to induction regimen.

Variable	Total ^1^	VCD *n* = 62 ^1^	D-VTD *n* = 37 ^1^	*p*-Value ^2^	*p*-Value ^3^
Age, years	55 (48–62)	57 (51–63)	52 (45–61)	0.111	
Sex, *n* (%)				0.005	0.693
Male	58 (58.6)	43 (69.4)	15 (40.5)		
Female	41 (41.4)	19 (30.6)	22 (59.5)		
MM subtype				0.304	
IgG	61 (62.9%)	36 (59.0%)	25 (69.4%)		
Non-IgG	36 (37.1%)	25 (41.0%)	11 (30.6%)		
ISS stage, *n* (%)				0.911	
I	31 (38.3)	19 (38)	12 (40)		
II	29 (36.3)	19 (38)	10 (33.3)		
III	20 (25)	12 (24)	8 (26.7)		
Cytogenetic risk, *n* (%)				0.272	
High risk	16 (16.2)	8 (12.9)	8 (21.6)		
Unknown	83 (83.8)	54 (87.1)	29 (78.4)		
Baseline GFR, *n* (%)				0.638	
GFR ≥ 30 mL/min	79 (85.9)	48 (87.3)	31 (83.8)		
GFR < 30 mL/min	13 (14.1)	7 (12.7)	6 (16.2)		
Baseline ECOG, *n* (%)				0.088	
<2	48 (52.7)	36 (59)	12 (40)		
≥2	43 (47.3)	25 (41)	18 (60)		
Lenalidomide maintenance, *n* (%)	71.7	39 (62.9)	32 (86.5)	0.012	0.350

^1^ Median (Q1–Q3); *n* (%); ^2^ Wilcoxon rank sum test; Pearson’s Chi-squared test; Fisher’s exact test; ^3^ Univariate Cox regression analysis for overall survival. Values are presented as *n* (%). Abbreviations: ECOG, Eastern Cooperative Oncology Group; GFR, glomerular filtration rate; IgG, immunoglobulin; ISS, International Staging System.

**Table 2:** Kaplan–Meier survival estimates and comparison by induction regimen.

(**A**) Kaplan–Meier estimates					
**Endpoint**	**Group**	**N**	**Events, *n* (%)**	**Median Survival, Months**	**95% CI, Months**
**OS**	Overall cohort	99	17 (17.2)	93	93–NE
	VCD	62	16 (25.8)	93	82–NE
	DVTD	37	1 (2.7)	NR	NE
**PFS**	Overall cohort	99	42 (42.4)	56	46–68
	VCD	62	36 (58.1)	56	44–68
	DVTD	37	6 (16.2)	40	37–NE
(**B**) Between-group comparison according to induction regimen					
**Endpoint**	**Comparison**	**HR**	**95% CI**	**Cox *p*-Value**	**Log-Rank *p*-Value**
**OS**	DVTD vs. VCD	0.18	0.02–1.38	0.099	0.06
**PFS**	DVTD vs. VCD	0.65	0.25–1.65	0.361	0.35

Abbreviations: OS, overall survival; PFS, progression-free survival; HR, hazard ratio; CI, confidence interval; NR, not reached; NE, not estimable; VCD, bortezomib-cyclophosphamide-dexamethasone; DVTD, daratumumab-bortezomib-thalidomide-dexamethasone.

**Table 3:** Treatment characteristics and responses following induction therapy.

Response Category	Total ^1^ N = 99 (%)	VCD ^1^, N = 62 (%)	D-VTD ^1^ N = 37 (%)	Odds Ratio ^3^ (95% CI)	*p*-Value ^2^	*p*-Value ^4^
Overall response	89 (89.9)	53 (85.5)	36 (97.3)	6.03 (0.78–274.91)	0.085	0.951
Complete response	36 (36.4)	15 (24.2)	21 (56.8)	4.12 (1.72–9.80)	0.002	0.620
VGPR or better	81 (81.8)	46 (74.2)	35 (94.6)	6.09 (1.31–28.23)	0.014	0.683
Partial response	17 (17.2)	15 (24.2)	2 (5.4)		0.025	0.347
Stable disease	1 (1.0)	1 (1.6)	0 (0.0)		1.000	
Progressive disease	9 (9.1)	8 (12.9)	1 (2.7)		0.147	

^1^ Median (Q1–Q3); *n* (%); ^2^ Wilcoxon rank sum test; Pearson’s Chi-squared test; Fisher’s exact test; ^3^ Odds ratio compares DVTD versus VCD; ^4^ Univariate analysis on overall survival.

**Table 4:** Response before and after autoSCT according to the induction regimen.

Response Assessment	Response Category	VCD	DVTD	*p* Value
**Before autoSCT**	CR	15/62 (24.2%)	21/37 (56.8%)	0.005
	VGPR	31/62 (50.0%)	14/37 (37.8%)	
	PR	15/62 (24.2%)	2/37 (5.4%)	
	SD	1/62 (1.6%)	0/37 (0%)	
**After autoSCT**	CR	28/61 (45.9%)	31/37 (83.8%)	0.002
	VGPR	23/61 (37.7%)	6/37 (16.2%)	
	PR	7/61 (11.5%)	0/37 (0%)	
	PD	3/61 (4.9%)	0/37 (0%)	
**Conversion after autoSCT among patients without CR before autoSCT**	non-CR to CR	18/47 (38.3%)	10/16 (62.5%)	0.092
	No conversion	29/47 (61.7%)	6/16 (37.5%)	

Abbreviations: autoSCT, autologous stem cell transplantation; CR, complete response; VGPR, very good partial response; PR, partial response; SD, stable disease; PD, progressive disease. *p* values are based on Pearson’s chi-square test. For the conversion analysis, Fisher’s exact test was *p* = 0.145.

**Table 5:** Patient characteristics according to EASIX calculated at diagnosis.

Variable	EASIX Group			
	EASIX < 3.74 (*n* = 82)	EASIX ≥ 3.74 (*n* = 10)	*p*-Value ^1^	*p*-Value ^2,3^
Age category			0.656	
<65	70 (85%)	8 (80%)		
≥65	12 (15%)	2 (20%)		
Sex			0.659	
Male	47 (57%)	5 (50%)		
Female	35 (43%)	5 (50%)		
MM subtype			0.087	
IgG	54 (68%)	4 (38.5%)		
Non-IgG	26 (33%)	6 (60%)		
ISS stage			0.002	---
I	30 (44.8%)	1 (10%)		
II	26 (38.8%)	2 (20%)		
III	13 (19%)	7 (70%)		
Cytogenetic high risk			0.818	
Yes	14 (18%)	2 (14%)		
Unknown	68 (83%)	8 (80%)		
Renal function (GFR)			<0.001	<0.001
>30 mL/min	77 (94.9%)	2 (20%)		
<30 mL/min	5 (6.1%)	8 (80%)		
ECOG category			0.840	
<2	39 (53)	5 (56)		
≥2	36 (48)	4 (44%)		
Induction therapy			0.504	
VCD	50 (61)	5 (50)		
D-VTD	32 (39)	5 (50)		
Lenalidomide maintenance			0.120	
Yes	63 (76.8)	5 (50.0)		
No	19 (23.2)	5 (50.0)		

^1^ Median (Q1–Q3); *n* (%); ^2^ Wilcoxon rank sum test; Pearson’s Chi-squared test; Fisher’s exact test; ^3^ Univariate analysis on overall survival. Values are presented as *n* (%). Abbreviations: EASIX, endothelial activation and stress index; ECOG, Eastern Cooperative Oncology Group; GFR, glomerular filtration rate; IgG, immunoglobulin; ISS, International Staging System; MM, multiple myeloma.

**Table 6:** Cutoff-based Kaplan–Meier results for overall survival according to the EASIX timing.

Parameter	EASIX at Diagnosis	Pre-AutoSCT EASIX
Patients included, *n*	92	99
Deaths, *n*	15	17
Censored patients, *n*	77	82
Selected log_2_(EASIX) cutoff	1.90	0.15
Original EASIX cutoff	3.74	1.11
Low-EASIX group, *n*	82	84
High-EASIX group, *n*	10	15
Deaths in low-EASIX group, *n*/N	9/82	10/84
Deaths in high-EASIX group, *n*/N	6/10	7/15
Median OS in low-EASIX group, months	93	Not reached
95% CI for median OS, low-EASIX group	93–not estimable	93–not estimable
Median OS in high-EASIX group, months	37	82
95% CI for median OS, high-EASIX group	21–not estimable	37–not estimable
Conventional log-rank *p*-value	<0.001	0.002
Exact-Gauss-adjusted maxstat *p*-value	<0.001	0.026
Conditional Monte Carlo-adjusted maxstat *p*-value	0.001	0.026

Abbreviations: EASIX, Endothelial Activation and Stress Index; OS, overall survival; autoSCT, autologous stem cell transplantation; CI, confidence interval.

**Table 7:** Cox regression analysis for overall survival and EASIX at diagnosis.

Model	Variable	HR (95% CI)	*p*-Value
**Univariable**	log_2_(EASIX) (continuous)	1.41 (1.15–1.72)	0.0007
	EASIX ≥ 3.74 vs. <3.74	7.64 (2.58–22.56)	0.0002
**Multivariable (main)**	log_2_(EASIX)	1.38 (1.11–1.71)	0.0035
	ISS II vs. I	1.20 (0.31–4.72)	0.79
	ISS III vs. I	1.77 (0.53–5.90)	0.36
**Multivariable (sensitivity)**	log_2_(EASIX)	1.36 (1.10–1.69)	0.0042
	ISS II vs. I	1.18 (0.30–4.68)	0.81
	ISS III vs. I	1.89 (0.56–6.35)	0.31
	Age (continuous)	1.02 (0.97–1.09)	0.42

The main multivariable model was adjusted for ISS stage, with additional adjustment for age in sensitivity analysis. EASIX was analyzed both as a continuous and dichotomized variable. The proportional hazards assumption was met for continuous models but not for the dichotomized EASIX variable. Abbreviation list: EASIX, Endothelial Activation and Stress Index; ISS, International Staging System.

**Table 8:** Cutoff-based Kaplan–Meier results for progression-free survival according to the EASIX timing.

Parameter	EASIX at Diagnosis	Pre-AutoSCT EASIX
Patients included, *n*	92	99
PFS events, *n*	39	42
Censored patients, *n*	53	57
Selected log_2_(EASIX) cutoff	1.53	−1.00
Original EASIX cutoff	2.88	0.50
Low-EASIX group, *n*	78	28
High-EASIX group, *n*	14	71
PFS events in low-EASIX group, *n*/N	31/78	12/28
PFS events in high-EASIX group, *n*/N	8/14	30/71
Median PFS in low-EASIX group, months (95% CI)	61 (51–71)	38 (24–not estimable)
Median PFS in high-EASIX group, months (95% CI)	17 (13–not estimable)	62 (54–72)
Maxstat statistic	3.03	2.32
Exact-Gauss-adjusted maxstat *p*-value	0.035	0.188
Conditional Monte Carlo-adjusted maxstat *p*-value	0.032	0.180

Abbreviations: EASIX, Endothelial Activation and Stress Index; PFS, progression-free survival; autoSCT, autologous stem cell transplantation; CI, confidence interval. Maxstat *p*-values are adjusted for outcome-based cutoff selection.

**Table 9:** Cox regression analyses of the EASIX for progression-free survival.

EASIX Timing	Model	EASIX Variable	HR	95% CI	*p*-Value
**Diagnosis**	Univariable	High vs. low EASIX	12.17	4.34–34.12	<0.001
		Continuous EASIX, per doubling	1.41	1.16–1.71	<0.001
	Adjusted	High vs. low EASIX	11.62	3.42–39.45	<0.001
		Continuous EASIX, per doubling	1.41	1.14–1.74	0.001
**Pre-autoSCT**	Univariable	High vs. low EASIX	0.29	0.13–0.66	0.003
		Continuous EASIX, per doubling	0.84	0.56–1.24	0.372
	Adjusted	High vs. low EASIX	0.21	0.09–0.51	<0.001
		Continuous EASIX, per doubling	0.74	0.47–1.18	0.204

Adjusted models included ISS stage, age, and sex. Continuous EASIX was analysed on the log_2_ scale; HRs therefore correspond to each doubling of the EASIX score.

## Data Availability

The original contributions presented in this study are included in the article. Further inquiries can be directed to the corresponding author.

## Abbreviations

The following abbreviations are used in this manuscript:

allo-HCT	Allogeneic hematopoietic cell transplantation
AUC	Area under the curve
autoSCT	Autologous stem cell transplantation
BM	Bone marrow
CA	Chromosomal abnormalities
CI	Confidence interval
C-index	Concordance index
COVID-19	Coronavirus disease 2019
CR	Complete response
D-VTd	Daratumumab, bortezomib, thalidomide, and dexamethasone
EASIX	Endothelial Activation and Stress Index
ECOG	Eastern Cooperative Oncology Group
FISH	Fluorescence in situ hybridization
GFR	Glomerular filtration rate
GVHD	Graft-versus-host disease
HR	Hazard ratio
IgG	Immunoglobulin G
IQR	Interquartile range
ISS	International Staging System
LDH	Lactate dehydrogenase
MGUS	Monoclonal gammopathy of undetermined significance
MLPA	Multiplex ligation-dependent probe amplification
MM	Multiple myeloma
NDMM	Newly diagnosed multiple myeloma
NE	Not estimable
NR	Not reached
NRM	Non-relapse mortality
OS	Overall survival
PD	Progressive disease
PFS	Progression-free survival
PR	Partial response
Q1	First quartile
Q3	Third quartile
R-ISS	Revised International Staging System
R2-ISS	Second Revision of the International Staging System
SCT	Stem cell transplantation
SD	Stable disease
SE	Standard error
SMM	Smoldering multiple myeloma
SPSS	Statistical Package for the Social Sciences
VCAM-1	Vascular cell adhesion molecule-1
VCD	Bortezomib, cyclophosphamide, and dexamethasone
VEGF	Vascular endothelial growth factor
VGPR	Very good partial response
